# Chronological Age Estimation of Male Occipital Bone Based on FTIR and Raman Microspectroscopy

**DOI:** 10.1155/2022/1729131

**Published:** 2022-08-26

**Authors:** Kai Yu, Hongli Xiong, Xin Wei, Hao Wu, Bo Zhang, Gongji Wang, Xiaorong Yang, Zhenyuan Wang

**Affiliations:** ^1^Department of Forensic Pathology, College of Forensic Medicine, Xi'an Jiaotong University, Xi'an 710061, China; ^2^Department of Forensic Medicine, Faculty of Basic Medical Sciences, Chongqing Medical University, Chongqing 400016, China; ^3^Xi'an Jiaotong University, Xi'an 710061, China; ^4^Department of Forensic Medicine, Guiyang Medical University, Guiyang, Guizhou 550025, China

## Abstract

Age-related changes in bone tissue have always been an important part of bone research, and age estimation is also of great significance in forensic work. In our study, FTIR and Raman microspectroscopy were combined to explore the structural and chronological age-related changes in the occipital bones of 40 male donors. The FTIR micro-ATR mode not only achieves the comparison of FTIR and Raman efficiency but also provides a new pattern for the joint detection of FTIR and Raman in hard tissue. Statistical analysis and PCA results revealed that the structure had little effect on the FTIR and Raman results. The FTIR and Raman mineral/matrix ratio, carbonate/phosphate ratio, crystallinity, and collagen maturity of the whole showed an increasing trend during maturation, and a significant correlation was found between FTIR and Raman by comparing four outcomes. Furthermore, the results indicated that the cutoff point of the change in the relative proportion of organic matrix and inorganic minerals in males was between 19 and 35 years old, and the changes in the relative proportion of organic matrix and inorganic minerals may play a key role in age estimation. Ultimately, we established age estimation regression models. The FTIR GA-PLS regression model has the best performance and is more suitable for our experiment (RMSECV = 10.405, RMSEP = 9.2654, *R*^2^CV = 0.814, and *R*^2^Pred = 0.828). Overall, FTIR and Raman combined with chemometrics are an ideal method to estimate chronological age based on age-dependent component changes in male occipital bones. Our experiment provides a proof of concept and potential experimental method for chronological age estimation.

## 1. Introduction

Bone is a complex hierarchical biological composite composed of inorganic minerals and organic fractions. Many studies have demonstrated that bone quality, in addition to bone mineral density (BMD), should also be considered when evaluating bone properties [1–3]. Bone quality is a broad term that encompasses factors affecting the morphology, microarchitecture, and material properties of bone. It is well known that bone quality changes during growth and maturation and is affected by external loads and hormone signals. For example, hydroxyapatite [Ca_10_(PO_4_)_6_(OH)_2_] constitutes most of the inorganic minerals in bone, which periodically deposits on soft organic matter to form mineral crystals and undergoes changes in crystal composition, perfection, and size during bone maturation [4, 5]. Type I collagen comprises 90% of the organic matrix in bone. Enzymatic crosslinks are formed within and between collagen fibers, and over time, they undergo a maturation process in which an immature divalent crosslink becomes a mature trivalent crosslink [6, 7]. Therefore, to explore the age-related changes in bone quality, high-resolution instruments are needed.

Fourier transform infrared spectroscopy (FTIR) and Raman spectroscopy are two different types of vibrational spectroscopy that are extremely powerful analytical techniques for the quantitative and qualitative analysis of components. Both techniques measure changes in the vibrational energies of intramolecular bonds excited by incident light, resulting in a spectrum that contains the “molecular fingerprint” of the sample [8]. FTIR and Raman spectroscopy are complementary techniques used to measure the changes in the molecular dipole moment and polarizability before and after vibration excitation, respectively. The combination of spectrometers with microscopes and focal plane array (FPA) detectors has given rise to FTIR and Raman microscopy and imaging techniques [9, 10]. Because of their high resolution, they are widely used to identify changes in bone material properties at the micron scale to assess disease [11], aging [12], and drug treatment [13]. Additionally, the adjacent molecular environment affects the vibrational features, thus providing additional information about the “molecular neighborhood.” The position, intensity, integral area, and width of the vibrational band can be used to monitor specific functional groups or regions of particular chemical species [14]. Recently, the development of new Raman techniques, such as spatial offset Raman spectroscopy (SORS) and tip-enhanced Raman spectroscopy (TERS), has provided technical support for the characterization of bone tissue composition in vivo and nanoscale bone properties, respectively [15]. Furthermore, over the past decade or so, tremendous effort has been made to develop small, portable, and user-friendly spectroscopic devices. These devices have provided significant benefits for a variety of applications, including forensics, medicine, military, security, and archaeological applications [16, 17]. With regard to forensic medicine in particular, evidence can be analyzed directly at a crime scene and in a nondestructive and confirmatory manner with the package [18].

Age estimation is a crucial part of forensic investigation. The accurate estimation of the chronological age of a victim or suspect can help investigators narrow the scope of search and improve efficiency in solving crimes. Bones or bone fragments can survive long postmortem degradation and may provide the only remaining information for a corpse. It is of great significance for forensic science to use bones or bone fragments for age estimation. In this study, FTIR and Raman microspectroscopy are combined to explore the structural and chronological age-related changes in the occipital bones of 40 male donors (with a chronological age of 0–74 years). It should be emphasized that, in this study, we chose to differentiate donors based on their chronological age rather than their biological age. This is because a person's biological age may be affected by factors such as health status (with or without disease), diet, and physical fitness. Therefore, two people who have the same chronological age may not appear to be the same biological age due to the above conditions [19]. The main purposes of our study were (1) to explore the structural differences of the occipital bone (lamina externa, diploe, lamina interna); (2) to explore the chronological age-related changes of the occipital bone; (3) to compare the results of FTIR and Raman bone research; and (4) to establish a chronological age estimation model.

## 2. Materials and Methods

### 2.1. Sample Preparation

All bone specimens in this study were jointly provided by the Forensic Judicial Expertise Center of Xi'an Jiaotong University and the Forensic Judicial Expertise Center of Guizhou Medical University, and the informed consent of relatives was acquired. It must be emphasized that all procedures for this study were in accordance with the requirements of local laws and institutional guidelines and were approved and supervised by the Ethics Committee of Xi'an Jiaotong University and Guizhou Medical University. None of the samples had a history of bone-related diseases. During the autopsy, a fragment of occipital bone approximately 3 cm × 3 cm was separated after the cranial vault was detached. Any soft tissue on the occipital bone fragments was removed with a scalpel, and then the bone fragments were rinsed with ultrapure water and fixed in 70% ethanol for 48 hours. Next, the bone fragments were modified to a suitable size by a small pendulum saw and attached to a slide with its cross section facing upward. The bone specimens were polished (EXAKT 400 CS, Germany) using silicon carbide paper with decreasing grit size (320, 500, 1000, 2500, and 4000 grit) in deionized water. The specimens were evaluated using an optical microscope to ensure surface smoothness. Finally, the specimens were rinsed again with ultrapure water and dried in an incubator at 40°C for 48 hours before testing. A total of 40 occipital bone specimens were collected from male donors with chronological ages ranging between 0 and 74 years (mean 33.92 years, standard deviation 23.71). To facilitate the comparison between different chronological age groups, the donors were split into six classes consisting of infants (0–3 years, *n* = 8), children (3–12 years, *n* = 2), adolescents (12–19 years, *n* = 3), early adults (19–35 years, *n* = 5), mid-adults (35–60 years, *n* = 17), and elderly adults (>60 years, *n* = 5).

### 2.2. Vibrational Spectroscopy

#### 2.2.1. FTIR Microspectroscopy

FTIR experiments were performed using a Bruker Spectrometer Vertex70 in microscopic attenuated total reflection (micro-ATR) mode. The spectrometer was equipped with a microscope in combination with a micro-20 × ATR accessory and liquid nitrogen-cooled FPA detector. The material of micro-ATR is Ge, and the number of reflections on the sample surface is 1 time. The FPA detector comprised 1024 pixels arranged in a 32 × 32 grid format. The spatial resolution of each pixel was 1.1 *μ*m × 1.1 *μ*m. The spectra were taken in the range from 3850 cm^−1^ to 400 cm^−1^ at a spectral resolution of 4 cm^−1^, with 32 scans for the sample spectra and 32 scans for the background spectra. In this study, 2 infrared images of different structures (lamina externa, diploe, and lamina interna) of each bone specimen were randomly collected, for a total of 6 infrared images. The sampling area of each infrared image is 35.2 *μ*m × 35.2 *μ*m, including 1024 spectra. The lamina externa and lamina interna of the occipital bone were marked when the samples were collected. Under the microscope, due to the special structure of the bone trabeculae, the diploe is obviously different from the lamina externa and lamina interna. The detection points of the lamina externa and lamina interna were selected, respectively, at the outer and inner edges of the bone, so as to prevent the detection points located at the junction area of different structures.

#### 2.2.2. FTIR Data Preprocessing

Preprocessing was carried out to improve the robustness and accuracy of subsequent data analysis and increase the interpretability of the spectral data [20]. After polynomial baseline correction (order = 2), each infrared image was subjected to a quality test. According to the maximum absorption intensity within the 1724–1475 cm^−1^ region, the upper and lower thresholds were defined as 0.3 and 0, respectively, and the spectra that did not meet the criteria were removed. Then, the spectral data were retained between the 800 cm^−1^ and 1800 cm^−1^ fingerprint regions and processed by standard normal variate (SNV). Spectra of the same structure (the reserved spectra of 2048 spectra after screening) were averaged to represent the spectral information of the structure of the bone specimen. The four widely used FTIR outcomes addressed in previous reviews were selected for analysis (Table 1). (1) The mineral/matrix ratio (area ratio of PO_4_^3−^*ν*_1_*ν*_3_/Amide I) measures the degree of mineralization of the collagen matrix [15, 21]. (2) The carbonate/phosphate ratio (area ratio of PO_4_^3−^*ν*_1_*ν*_3_/CO_3_^2−^*ν*_2_) measures the degree of carbonate substitution into the mineral crystal lattice at the A OH^−^, B PO_4_^3−^, and labile positions [5, 22]. (3) Crystallinity (intensity ratio of 1030 cm^−1^/1020 cm^−1^) is a complex outcome reflecting both crystal size (thickness and length) and stoichiometric perfection (chemical composition and structural order) [5, 15]. (4) Collagen maturity measures the secondary structure of the collagen fibers and is calculated as the intensity ratio of mature trivalent crosslinks (predominantly pyridinoline (Pyr) at 1660 cm^−1^) to immature divalent crosslinks (predominantly dehydro-dihydroxylysinonorleucine (DHLNL) at 1690 cm^−1^) [6, 7]. Moreover, the spectral data were also analyzed by mean centering during partial least-squares (PLS) regression algorithm analysis.

#### 2.2.3. Raman Microspectroscopy

Raman spectra of the bone specimens detected by Raman were recorded using an inVia Renishaw spectrometer. The spectrometer was equipped with a Leica microscope in combination with a 50x objective and CCD detector. The 785 nm excitation laser source was used to collect Raman spectra in the reflection mode for the frequency region from 2000 cm^−1^ to 300 cm^−1^ with a resolution of 1 cm^−1^ and a laser spot size of 1 *μ*m. The laser was operated at 100% power (250 mW at the laser exit) with a 10 s exposure time and 3 accumulation times. Under these parameters, better signal-to-noise ratio can be obtained without damaging samples. In this study, 3 spectra of different structures (lamina externa, diploe, and lamina interna) of each bone specimen were randomly collected, for a total of 9 spectra.

#### 2.2.4. Raman Data Preprocessing

The Raman spectra were denoised by Savitzky–Golay (SG) smoothing (11 points of smoothing, order = 3), and the light scattering was corrected by SNV [23]. Cosmic spikes were removed, and the fluorescence effect was subtracted by baseline correction by fitting a third-degree polynomial curve iteratively to the spectra. Spectra of the same structure (3 spectra) were averaged to represent the spectral information of the structure of the bone specimen. The four Raman outcomes corresponding to FTIR outcomes were selected for analysis (Table 1). (1) Mineral/matrix ratio: the peak area ratios PO_4_^3−^*ν*_2_/amide III were selected to minimize the effects of orientation [10, 24, 25]. (2) Carbonate/phosphate ratio: the peak area ratio of the composite CO_3_^2−^*ν*_1_–PO_4_^3−^*ν*_3_/PO_4_^3−^*ν*_1_ peaks [10, 25]. (3) Crystallinity: the inverse of the full-width half-maximum (FWHM) of the PO_4_^3−^*ν*_1_ peak in the Raman spectra [26]. (4) Collagen maturity: the intensity ratio of 1660 cm^−1^/1690 cm^−1^ [27]. Moreover, the spectral data were also analyzed by mean centering during partial least-squares (PLS) regression algorithm analysis.

### 2.3. Statistical Analysis

The outcomes of FTIR and Raman were statistically analyzed using SPSS (version 24.0, IBM, Armonk, New York). Single-factor ANOVA was used to compare the parameters of bone structure and chronological age, and the Student–Newman–Keuls (SNK) test was used for pairwise comparisons. In groups that failed to exhibit normal distributions or equal variance, the Kruskal–Wallis test was used to compare the parameters of bone structure and chronological age, and the nonparametric Mann–Whitney *U* test was used for pairwise comparisons. Additionally, Pearson's correlation test was used to compare the parameters of FTIR and Raman spectroscopy. A *P* value < 0.05 was considered to be statistically significant.

### 2.4. Chemometrics and Software

Principal component analysis (PCA) is a mathematical algorithm used for dimensionality reduction [28]. The algorithm extracts a group of correlated spectral variables and transforms them into a group of smaller unrelated variables referred to as principal components (PCs). The loadings as the coefficients of the corresponding PCs are often used to account for the contribution of variables in PCs. In this study, PCA was performed on the FTIR and Raman spectral data of different structures (lamina externa, diploe, and lamina interna) of the occipital bones of 40 male donors.

Partial least-squares (PLS) regression is a supervised multiple regression method for quantitative analysis [29, 30]. By extracting latent variables (LVs), PLS can construct a predictive model between a set of explanatory variables *X* and a response *Y* (the spectral data matrix and chronological age in this study). For the PLS algorithm, the determination of LVs is a crucial step to optimize the prediction ability of the model, and it usually relies on cross-validation (CV). Therefore, Venetian blind 20-fold CV was performed to select the best number of LVs and build the model in this study. The genetic algorithm (GA) is a mathematical optimization method inspired by species evolution according to Darwin's theory of the “survival of the fittest” [31]. The GA is applied to feature selection of the spectral dataset to help identify a subset of the measured variables and build a more accurate and precise regression model. The calibration model was established using the effective variables selected by GA-PLS, and the efficiency was compared with the PLS reference model using all variables in a certain spectral range.

Before establishing PLS and GA-PLS regression models of chronological age, the FTIR (*n* = 120) and Raman (*n* = 120) spectral data of three structures (lamina externa, diploe, and lamina interna) of the occipital bones of 40 male donors were divided into a calibration dataset (70%, *n* = 84) and prediction dataset (30%, *n* = 36) by K-Stone sampling. It is worth mentioning that the samples in the prediction dataset did not undergo variable selection or calibration procedures. The root mean square error of cross-validation (RMSECV), root mean square error of prediction (RMSEP), determination coefficient of cross-validation (*R*^2^CV), and determination coefficient of prediction (*R*^2^Pred) were used to evaluate the two regression models. RMSECV usually represents the magnitude of the global model error in the calibration model, while RMSEP is used to evaluate the performance of the prediction dataset. Lower RMSECV and RMSEP, but small differences between these two values, indicate better predictive quality. Furthermore, the determination coefficient (*R*^2^) can be used to evaluate the degree of fit between actual and predicted values. The closer it is to 1, the better the model fits. The variable importance in projection (VIP) was calculated for all spectral variables to evaluate the contribution explaining the distinction in the PLS regression model [32].

All data were analyzed using MATLAB R2019b (The MathWorks, MA, USA) equipped with PLS Toolbox 8.7.1 (Eigenvector Research, WA, USA).

## 3. Results

### 3.1. FTIR and Raman Average Preprocessed Spectra

**Figure 1:**
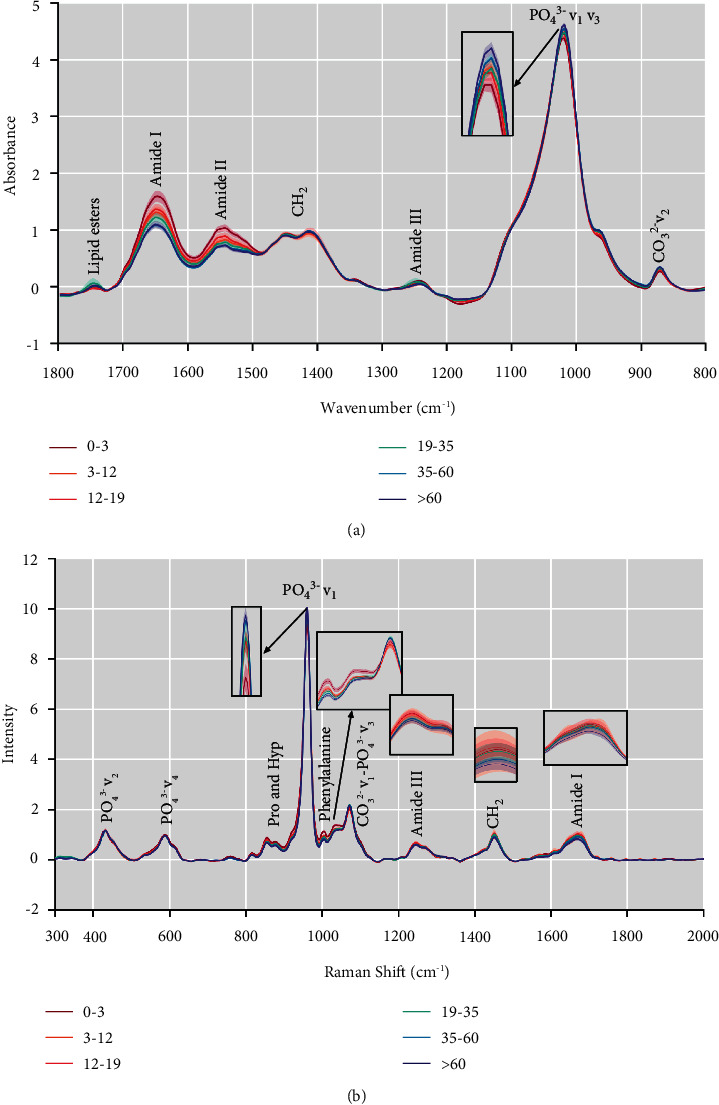
Average preprocessed spectra of FTIR (a) and Raman (b) at 6 age stages. The lines correspond to the average of the spectral dataset, and the shaded area represents the 95% confidence region of the variability of the dataset. Primary band assignments in the FTIR and Raman spectra of bone were marked in the figure.

The average preprocessed FTIR (Figure 1(a)) and Raman (Figure 1(b)) spectra at 6 age stages are shown in Figure 1. The strongest FTIR absorption band is at 1200–900 cm^−1^, which is attributed to PO_4_^3−^*ν*_1_*ν*_3_. The regions of 1592–1712 cm^−1^ and 1510–1590 cm^−1^ are assigned to amide I and amide II, respectively. The CO_3_^2−^*ν*_2_ substituted for the apatite lattice has an FTIR absorption peak at 850–890 cm^−1^, consisting of type A, which denotes carbonate in the hydroxyl position of apatite (A OH^−^), type B carbonate in phosphate (B PO_4_^3−^), and labile, which signifies loosely adsorbed carbonate on the crystal surfaces (labile positions). The PO_4_^3−^*ν*_1_ stretching vibration at 930–980 cm^−1^ is the strongest sign of Raman bone mineral, while the PO_4_^3−^*ν*_2_ and PO_4_^3−^*ν*_4_ bending vibrations are at 410–460 cm^−1^ and 587 cm^−1^, respectively. CO_3_^2−^*ν*_1_–PO_4_^3−^*ν*_3_ has a prominent composite band at 1050–1100 cm^−1^. In addition, the bands observed in the high-frequency region are *ν*(C–C) phenylalanine (1003 cm^−1^), amide III (1215–1300 cm^−1^), the C–H bending mode (1446 cm^−1^), and amide I (1620–1700 cm^−1^). More detailed FTIR and Raman primary band assignments are shown in Table 1 and Figure 1. In Figure 1(a), the intensity of amide I and amide II decreased with age, while the intensity of PO_4_^3−^*ν*_1_*ν*_3_ increased with age. In Figure 1(b), the intensity of phenylalanine, amide III, CH_2_ and amide I decreased with age, while the intensity of PO_4_^3−^*ν*_1_ and CO_3_^2−^*ν*_1_–PO_4_^3−^*ν*_3_ increased with age.

### 3.2. FTIR and Raman Outcomes

**Figure 2:**
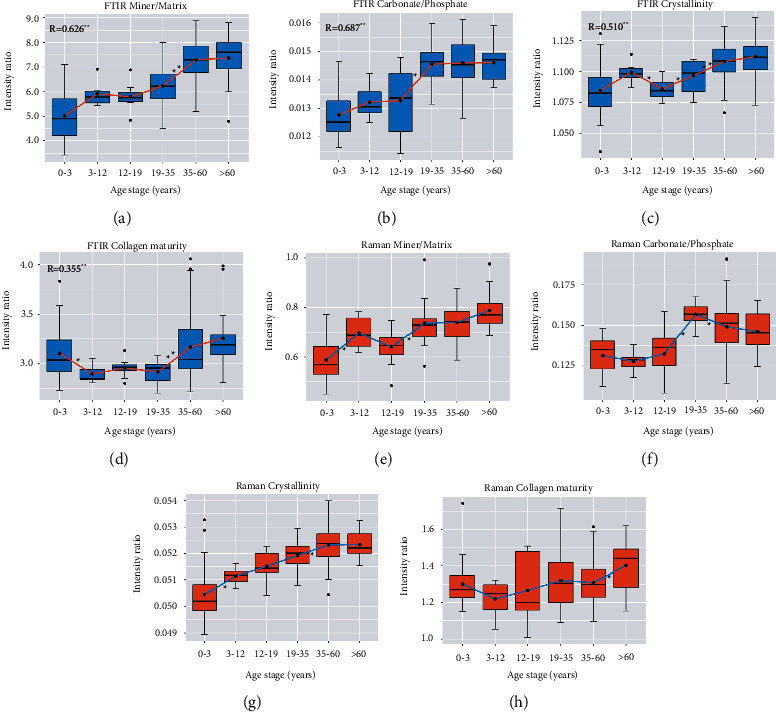
Four outcomes at 6 age stages were evaluated by FTIR and Raman and displayed by box and mean line plots. (a) FTIR mineral/matrix ratio (900–1200 cm^−1^/1592–1712 cm^−1^). (b) FTIR carbonate/phosphate ratio (850–890 cm^−1^/900–1200 cm^−1^). (c) FTIR crystallinity (1030 cm^−1^/1020 cm^−1^). (d) FTIR collagen maturity (1660 cm^−1^/1690 cm^−1^). (e) Raman mineral/matrix ratio (410–460 cm^−1^/1215–1300 cm^−1^). (f) Raman carbonate/phosphate ratio (1050–1100 cm^−1^/930–980 cm^−1^). (g) Raman crystallinity (1/FWHM PO_4_^3−^*ν*_1_). (h) Raman collagen maturity (1660 cm^−1^/1690 cm^−1^). Significant differences are indicated, ^*∗*^*P* < 0.05, ^*∗∗*^*P* < 0.01 (SNK or Mann–Whitney *U*-test). Pearson correlation coefficients *R* between the techniques are indicated in (a)–(d).

First, the FTIR and Raman four outcomes of different structures (lamina externa, diploe, and lamina interna) of each age stage were analyzed. Only the carbonate/phosphate ratio of Raman in 19–35 was statistically significant (*P*=0.002), while SNK revealed that the lamina externa and lamina interna (*P* = 0.017) and the diploe and lamina interna (*P*=0.001) were statistically significant (Table S1). Therefore, in the age-stage single-factor ANOVA, the lamina interna carbonate/phosphate ratio of Raman in 19–35 was discarded. Based on the above results, we find that the structure hardly affects the FTIR and Raman outcomes. Then, statistical analysis was performed for each age range. Both FTIR and Raman techniques revealed significant changes in bone composition during maturation (Table 2 and Figure 2). A significant correlation was found between FTIR and Raman by comparing four outcomes (Figure 2): mineral/matrix ratio (*R* = 0.626, *P* < 0.01), carbonate/phosphate ratio (*R* = 0.687, *P* < 0.01), crystallinity (*R* = 0.510, *P* < 0.01), and collagen maturity (*R* = 0.335, *P* < 0.01). FTIR and Raman mineral/matrix ratios generally increased during maturation (Figures 2(a) and 2(e)). The FTIR and Raman carbonate/phosphate ratios also increased as a whole and increased significantly from 12 to 19 to 19–35 (Figures 2(b) and 2(f)). The crystallinity was found to increase throughout maturation in the Raman spectra (Figure 2(g)), while the FTIR crystallinity fluctuated between 0 and 19 years, but the overall TIR crystallinity also increased (Figure 2(c)). The collagen maturity of FTIR and Raman showed a similar trend of first decreasing and then gradually increasing (Figures 2(d) and 2(h)). However, infrared collagen maturity was statistically significant, while Raman was not (*P*=0.094).

### 3.3. Characterization of FTIR and Raman by PCA

**Figure 3:**
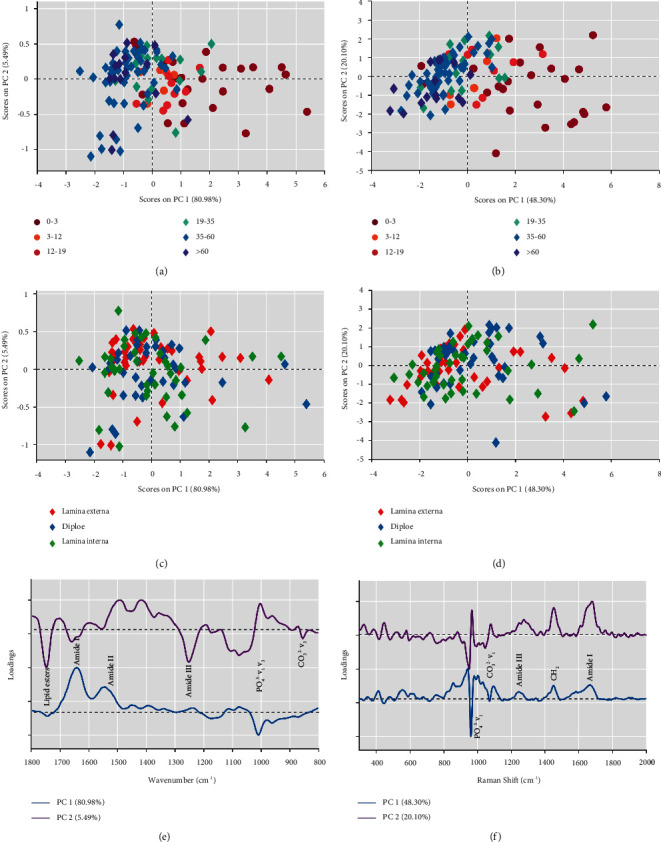
The PCA results of FTIR and Raman. (a) FTIR PCA score plot for 6 age stages. (b) Raman PCA score plot for 6 age stages. (c) FTIR PCA score plot for 3 structures (lamina externa, diploe, and lamina interna). (d) Raman PCA score plot for 3 structures (lamina externa, diploe, and lamina interna). (e) FTIR PC1 and PC2 loading plot. (f) Raman PC1 and PC2 loading plot.

PCA was used to analyze the FTIR and Raman spectral datasets. In Figure 3(a), the two-dimensional distribution of samples is displayed, and a great splitting trend between 0 and 19 years and >19 years is observed along PC1, which explains 80.98% of the total variation. There is no good differentiation in the direction of PC2. Combined with the PC1 loading plot in Figure 3(e), the negative correlation loading representing >19 years primarily appears in PO_4_^3−^*ν*_1_*ν*_3_, while the positive correlation loading representing 0–19 years mainly appears in amide I and amide II. The same results were found in the Raman PCA score plot (Figure 3(b)). The loading plot of PC1 (explaining 48.3% of the total variation) demonstrates that the negative correlation loading representing >19 years primarily appears in PO_4_^3−^*ν*_1_, while the positive correlation loading representing 0–19 years mainly appears in amide III, CH_2_, and amide I (Figure 3(f)). Furthermore, the PCA results of both FTIR and Raman revealed that the samples representing lamina externa, diploe, and lamina interna clustered with each other and without a separation trend (Figures 3(c) and 3(d)). Eight widely used FTIR and Raman outcomes were also analyzed by PCA, and PC1 explained 94.29% of the total variation.  Figure S1A shows that samples moved from the negative axis to the positive axis along PC1 with increasing age. The FTIR mineral/matrix ratio was found to be the main contribution in the PC1 loading plot (Figure S1B).

### 3.4. Estimate Chronological Age by PLS and GA-PLS

**Figure 4:**
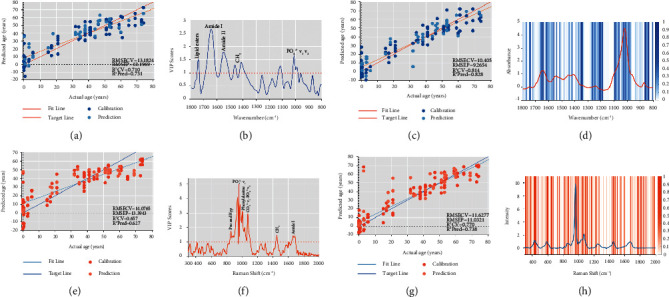
PLS and GA-PLS regression models of FTIR and Raman age estimation. The dark color dots represent the calibration datasets, and the light color dots represent the prediction datasets. The dark color line is the target line corresponding to the perfect prediction, while the light color line is the model fitting line. (a) FTIR PLS regression model. (b) FTIR PLS regression model VIP scores plot. (c) FTIR GA-PLS regression model. (d) FTIR variables selected by GA (blue shades). (e) Raman PLS regression model. (f) Raman PLS regression model VIP scores plot. (g) Raman GA-PLS regression model. (h) Raman variables selected by GA (orange shades).

Our next step was to establish a PLS regression model and GA-PLS regression model to estimate chronological age. The detailed results of the model performance are summarized in Table 3. First, we built the PLS regression model with the eight outcomes of FTIR and Raman. As illustrated in Figure S2A, the abscissa is the actual age, and the ordinate represents the predicted age. The cyan dots represent the calibration datasets, and the light cyan dots represent the prediction datasets. The *R*^2^ of the internal CV (calibration dataset) was only 0.474, and that of the prediction dataset was only 0.383. The VIP score plot shown in Figure S2B was used to evaluate the importance of the variables. The variable with higher VIP scores was the FTIR mineral/matrix ratio. Subsequently, we established the PLS and GA-PLS regression models of FTIR and Raman age estimation, respectively. Figures 4(a) and 4(b) show the FTIR PLS regression model and its corresponding VIP score plot, respectively. The RMSECV and *R*^2^ of internal CV are 13.1824 and 0.710, respectively, and the RMSEP and *R*^2^ of the prediction dataset are 12.1969 and 0.731, respectively. The variables with higher VIP values were lipid ester, amide I, amide II, CH_2_, and PO_4_^3−^*ν*_1_*ν*_3_. Figures 4(e) and 4(f) show the Raman PLS regression model (RMSECV = 14.0765, RMSEP = 13.3943, *R*^2^CV = 0.657, *R*^2^Pred = 0.627) and its corresponding VIP score plot. The variables with higher VIP values were proline, hydroxyproline, PO_4_^3−^*ν*_1_, phenylalanine, CO_3_^2−^*ν*_1_–PO_4_^3−^*ν*_3_, CH_2_, and amide I. GA was used to extract significant spectral variables. The parameters used for optimization are as follows: population size, 50 chromosomes; window width, FTIR is 1 and Raman is 8; max generations, 200; mutation rate, 0.01; crossover, double; replicate runs, 50. A total of 32 variables and 196 variables were ultimately selected by FTIR and Raman spectroscopy, respectively, and are displayed with blue shadows and orange shadows in Figures 4(d) and 4(h), respectively. The darker the shadow color is, the higher the frequency of selecting spectral variables in the iterative process is, and the more important it is for the establishment of the regression model. As shown in the figure, the selected variables mainly exist in the position of the wave peak, which is almost consistent with the variables selected by the VIP score plot. Finally, according to the spectral variables extracted by GA, the GA-PLS regression models of FTIR (RMSECV = 10.405, RMSEP = 9.2654, *R*^2^CV = 0.814, *R*^2^Pred = 0.828) and Raman (RMSECV = 11.6277, RMSEP = 11.0321, *R*^2^CV = 0.770, *R*^2^Pred = 0.738) were established.

## 4. Discussion

In the present study, FTIR and Raman spectroscopy were combined to explore the structural and chronological age-related changes in the occipital bones of 40 male donors, and FTIR and Raman age estimation models were established. FTIR experiments were performed in micro-ATR mode. It is important to emphasize the advantages of using the micro-20 × ATR accessory. The ATR probe is in direct contact with the bone specimen when obtaining a high-resolution spectral image of the contact surface. The preparation of bone specimens in the traditional transmission mode requires bones to be embedded in an infiltrating plastic resin, which may change the chemical composition and cause spectral overlap with the absorption properties of bone [33]. In addition, thin sections of bone (<5 *μ*m) are needed, as specimens that are too thick will cause insufficient illumination of the detector, affecting accurate measurements and making quantification impossible [34]. Compared with transmission mode, micro-ATR mode only requires simple sample pretreatment to perform FTIR and Raman detection on identical bone specimens. It not only realizes the comparison of FTIR and Raman efficiency, but also provides a new pattern for the joint detection of FTIR and Raman in hard tissue.

Skull bones such as frontal, temporal, parietal, and occipital bones are similar to a “Sandwich” structure, consisting of the lamina externa, diploe, and lamina interna [35]. The lamina externa and lamina interna are osteon-dense bone, and the diploe is cancellous bone. The osteon-dense bone consists of a compact and regularly arranged osteon, while the cancellous bone consists of trabecular bone. The results of statistical analysis (Table 1) and PCA (Figures 3(c) and 3(d)) revealed that the structure had little effect on the FTIR and Raman results, which may be because FTIR and Raman are used to characterize the changes in the chemical composition of bone, while trabecular bone is the link between lamina externa and lamina interna, so the lamina externa, diploe, and lamina interna have similar compositions. This is not contradictory to the different microstructures and biomechanical properties of osteon-dense bone and cancellous bone [36, 37]. Previous FTIR microspectroscopy and imaging studies showed reproducible patterns for bone mineral properties across cortical osteons and along forming trabecular surfaces from normal human iliac crest biopsies [8, 9], which is consistent with our results. Moreover, PCA also verified that the difference in age was much greater than that in structure (Figure 3). Therefore, in the follow-up analysis, we no longer considered the interference of the structure but only investigated age-related changes.

Four corresponding FTIR and Raman outcomes related to bone quality were compared during the maturation of bone. The FTIR and Raman mineral/matrix ratio, carbonate/phosphate ratio, crystallinity, and collagen maturity of the whole sample showed an increasing trend during maturation. There is a rapid rise phase before the age of 35, which then reaches a plateau. The mineral/matrix ratio is a measure of bone mineral density, which has been shown to correlate with the measurements of bone ash weight [38]. The increase in the mineral/matrix ratio with age has been confirmed by numerous animal and human bone studies (Figures 2(a) and 2(e)) [12, 25, 39]. There are three substitutional types of carbonates, including A OH^−^, B PO_4_^3^, and labile positions [40]. The FTIR carbonate/phosphate ratio measures the degree of substitution of the three types, while the Raman carbonate/phosphate ratio only measures the degree of substitution of type B carbonate. With increasing age, the FTIR and Raman carbonate/phosphate ratios increased and increased significantly from 12 to 19 to 19–35 (Figures 2(b) and 2(f)) and then stabilized gradually. This phenomenon seems to imply a peak of carbonate substitution between 19 and 35 years old, which coincides with the report by Van [41] and Hoiberg [42] et al. that the timing of peak bone mass occurred between the second to third decade life for males, followed by a plateau. Crystals initially nucleate on collagen fibers as disordered calcium phosphate precipitates. Over time, these precipitates become larger and more perfectly structured, forming a platelet-shaped hydroxyapatite-like carbonate-substituted calcium phosphate. Therefore, FTIR and Raman crystallinity increase with tissue age [43], which is consistent with our results (Figures 2(c) and 2(g)). The collagen maturity of FTIR and Raman showed a similar trend of first decreasing and then gradually increasing (Figures 2(d) and 2(h)). Over time, the enzymatic crosslinks in collagen undergo an immature divalent crosslink (predominantly DHLNL at 1690 cm^−1^) to a mature trivalent crosslink (predominantly Pyr at 1660 cm^−1^) [6, 7]. Therefore, collagen maturity increases with age. However, Pyr and DHLNL are only two of the major collagen crosslinks, so the temporal and spatial distribution of collagen properties and their relationship with bone are only partially related. Infant (0–3 years) bone contains a high proportion of specific collagen, which may be the reason why the FTIR and Raman collagen maturity first decreases and then gradually increases. Additionally, a significant correlation was found between FTIR and Raman by comparing four outcomes (Figure 2), indicating that FTIR and Raman reflect similar changes in bone chemical composition.

Regarding eight FTIR and Raman outcomes, FTIR and Raman PCA results all showed that samples moved from the negative axis to the positive axis along PC1 with the increase of age stage, with a clear age cutoff point between 19 and 35 years old, which again seemed to support the timing of peak bone mass occurring between the second to third decade life for males (Figure S1A, Figures 3(a) and 3(b)). Surprisingly, combined with the respective PC1 loading plots of the three PCA results, we found contributions not only from inorganic minerals, but also from the organic matrix (Figure S1B, Figures 3(e) and 3(f)). The 0–19-year group had a higher proportion of organic matrix bands, including amide I and amide II from FTIR and amide III, CH_2_, and amide I from Raman. In contrast, the >19 years has a higher proportion of inorganic mineral bands, including PO_4_^3−^*ν*_1_*ν*_3_ from FTIR and PO_4_^3−^*ν*_1_ from Raman. The outcome also indicated that the increase in the mineral/matrix ratio with age is the common result of an increase in minerals and a decrease in the organic matrix. The same phenomenon could be found more clearly in the average spectrogram (Figures 1(a) and 1(b)). Therefore, we speculated that the cutoff point of the change in the relative proportion of organic matrix and inorganic minerals in males was between 19 and 35 years old. Ultimately, we established eight FTIR and Raman outcomes and FTIR and Raman regression models. The model established by eight FTIR and Raman outcomes is not ideal, indicating that the locally extracted spectral variables cannot support the establishment of the model (Table 3). The application of GA not only significantly reduces the number of variables, but also improves the performance of the model. This demonstrates that the GA can effectively select relatively informative variables and eliminate useless variables or even noise. This is extremely important for model maintenance because it is well known that simpler models are more robust and less sensitive to random variability [44]. The overall performance of the FTIR model is better than that of the Raman model (Table 3), probably because the signal-to-noise ratio of FTIR is higher than that of Raman, so the differences between variables can be better captured by the algorithm [45]. Therefore, the FTIR GA-PLS regression model has the best performance and is more suitable for our experiment. In addition, the variables selected by PCA loading plots, VIP score plots (Figures 4(b) and 4(f)), and GA (Figures 4(d) and 4(h)) seem to reveal that changes in the relative proportion of organic matrix and inorganic minerals may play a key role in age estimation.

## 5. Conclusion

In our study, FTIR and Raman spectroscopy were combined to explore the structural and chronological age-related changes in the occipital bone of 40 male donors. FTIR and Raman combined with chemometrics are an ideal method to estimate chronological age based on age-dependent component changes in male occipital bones. The results indicate that the cutoff point of the change in the relative proportion of organic matrix and inorganic minerals in males was between 19 and 35 years old, and the changes in the relative proportion of organic matrix and inorganic minerals may play a key role in age estimation. Finally, PLS and GA-PLS regression models of FTIR and Raman were established for age estimation, and the FTIR GA-PLS regression model had the best performance. However, more work needs to be performed before this new approach can be employed in practice. For example, the effects of individual differences (sex, disease history, postmortem interval, etc.) and bone types (long, short, flat, and irregular bones) on spectral results were not taken into consideration in our study. In addition, more samples need to be collected to improve the robustness and accuracy of the model. Nevertheless, this experiment provides a proof-of-concept and potential experimental method for chronological age estimation.

## Figures and Tables

**Table 1:** Primary band assignments in the FTIR and Raman spectrum of bone and the four widely used outcomes.

Parameter	FTIR (cm^−1^)	Raman (cm^−1^)
PO_4_^3−^*ν*_2_	—	410–460
PO_4_^3−^*ν*_4_	—	587
*ν*(C–C) hydroxyproline	—	874
*ν*(C–C) proline	—	920
CO_3_^2−^*ν*_2_	850–890	—
PO_4_^3−^*ν*_1_	—	930–980
PO_4_^3−^*ν*_1_*ν*_3_	900–1200	—
*ν*(C–C) phenylalanine	—	1003
CO_3_^2−^*ν*_1_–PO_4_^3−^*ν*_3_	—	1050–1100
Amide III	1210–1320	1215–1300
CH_2_	1410, 1445	1446
Amide II and CO_3_^2−^*ν*_3_	1510–1590	—
Amide I	1592–1712	1620–1700
Lipid esters	1745	—
Mineral/matrix	(900–1200) : (1592–1712)	(410–460) : (1215–1300)
Carbonate/phosphate	(850–890) : (900–1200)	(1050–1100) : (930–980)
Crystallinity	1030 : 1020	1 : FWHM PO_4_^3−^*ν*_1_
Collagen maturity	1660 : 1690	1660 : 1690

**Table 2:** FTIR and Raman statistical results in different age stage. The values are represented as mean ± SD. ^*∗*^The results of Kruskal–Wallis test that failed to exhibit normal distributions or equal variance. And the rest are the results of single-factor ANOVA.

Parameter	0∼3 years	3∼12 years	12∼19 years	19∼35 years	35∼60 years	>60 years	*P*-Value
FTIR							
Mineral/matrix	5.0219 ± 1.1423	5.9182 ± 0.5449	5.8013 ± 0.547	6.2286 ± 1.0708	7.2973 ± 0.8775	7.3667 ± 0.1226	<0.01^*∗*^
Carbonate/phosphate	0.0128 ± 0.0008	0.0132 ± 0.0006	0.0133 ± 0.0011	0.0146 ± 0.0008	0.0146 ± 0.0008	0.0146 ± 0.0006	<0.01^*∗*^
Crystallinity	1.0847 ± 0.0230	1.0992 ± 0.0089	1.0859 ± 0.0082	1.0965 ± 0.0126	1.1080 ± 0.0158	1.1120 ± 0.0173	<0.01
Collagen maturity	3.1074 ± 0.2716	2.8954 ± 0.0969	2.9560 ± 0.0946	2.9176 ± 0.1182	3.1644 ± 0.3339	3.2543 ± 0.3389	<0.01^*∗*^

Raman							
Mineral/matrix	0.5880 ± 0.0899	0.6980 ± 0.0692	0.6417 ± 0.0814	0.7369 ± 0.0977	0.7388 ± 0.0692	0.7884 ± 0.0775	<0.01
Carbonate/phosphate	0.1315 ± 0.0102	0.1280 ± 0.0069	0.1326 ± 0.0165	0.1569 ± 0.0073	0.1492 ± 0.0141	0.1461 ± 0.0124	<0.01
Crystallinity	0.0504 ± 0.0011	0.0511 ± 0.0003	0.0515 ± 0.0006	0.0519 ± 0.0005	0.0523 ± 0.0006	0.0523 ± 0.0005	<0.01^*∗*^
Collagen maturity	1.3023 ± 0.1286	1.2197 ± 0.1054	1.2662 ± 0.1815	1.3219 ± 0.1631	1.3099 ± 0.1176	1.4021 ± 0.1462	0.094^*∗*^

**Table 3:** Results of PLS and GA-PLS regression model of chronological age estimation. The optimized models are shown in bold.

Instrument	Method	VNs	LVs	Cross-validation		Prediction	
				*R* ^2^CV	RMSECV	*R* ^2^Pred	RMSEP
Eight outcomes	PLS	8	1	0.474	16.1257	0.383	20.4607
FTIR	PLS	259	8	0.710	13.1824	0.731	12.1969
	GA-PLS	**32**	**9**	**0.814**	**10.405**	**0.828**	**9.2654**
Raman	PLS	1557	4	0.657	14.0765	0.627	13.3943
	GA-PLS	**196**	**10**	**0.770**	**11.6277**	**0.738**	**11.0321**

VNs: variable numbers; LVs: latent variables; *R*^2^CV: determination coefficient of cross-validation; RMSECV: root mean square error of cross-validation; *R*^2^Pred: determination coefficient of prediction; RMSEP: root mean square error of prediction.

## Data Availability

The data that support the findings of this study are available upon request from the corresponding author.

## Supplementary Materials

Supplementary Materials Table S1: FTIR and Raman statistical results of different structures (lamina externa, diploe, and lamina interna) at each age stages. ^*∗*^The results of Kruskal–Wallis test that failed to exhibit normal distributions or equal variance. And the rest are the results of Single-factor ANOVA. Figure S1: The PCA results of eight widely used FTIR and Raman outcomes. A PCA score plot for 6 age stages. B PC1 loading plot. Figure S2: A PLS regression model with the eight outcomes of FTIR and Raman. B PLS regression model VIP scores plot.
