# Supplementary material for: Chronological Age Estimation of Male Occipital Bone Based on FTIR and Raman Microspectroscopy
Source: Bioinorg Chem Appl. 2022 Aug 26;2022:1729131. doi: 10.1155/2022/1729131 (PMC9440630; doi:10.1155/2022/1729131)
Supplement: Supplementary Materials — Table S1: FTIR and Raman statistical results of different structures (lamina externa, diploe, and lamina interna) at each age stages. ∗The results of Kruskal–Wallis test that failed to exhibit normal distributions or equal variance. And the rest are the results of Single-factor ANOVA. Figure S1: The PCA results of eight widely used FTIR and Raman outcomes. A PCA score plot for 6 age stages. B PC1 loading plot. Figure S2: A PLS regression model with the eight outcomes of FTIR and Raman. B PLS regression model VIP scores plot. [file 1729131.f1.zip › 1729131.f1/Supplementary Material .docx]

Chronological age estimation of male occipital bone based on FTIR and Raman microspectroscopy

Kai Yu^1^, Hongli Xiong^3^, Xin Wei^1^, Hao Wu^1^, Bo Zhang^4^, Gongji Wang^1^, Xiaorong Yang^2^*, Zhenyuan Wang^1^*

^1^Department of Forensic Pathology, College of Forensic Medicine, Xi’an Jiaotong University, Xi’an 710061, PR China

^2^Department of Forensic Medicine, Guiyang Medical University, Guiyang, Guizhou, 550025, PR China

^3^ Department of Forensic Medicine, Faculty of Basic Medical Sciences, Chongqing Medical University, Chongqing 400016, PR China

^4^ Xi’an Jiaotong University, Xi’an 710061, PR China

*Corresponding author: Zhenyuan Wang, PhD, Department of Forensic Pathology, College of Forensic Medicine, Xi’an Jiaotong University, Xi’an 710061, PR China. Phone: +8629 13709110298. E-mail: wzy218@xjtu.edu.cn.

Xiaorong Yang, MD, Department of Forensic Medicine, Guiyang Medical University, Guiyang, Guizhou, 550025, PR China. Phone: +86851 18085148797. E-mail: fyyxr_820613@163.com.

Table S1 FTIR and Raman statistical results of different structures (lamina externa, diploe and lamina interna) at each age stages. *The results of Kruskall-Wallis test that failed to exhibit normal distributions or equal variance. And the rest are the results of Single-factor ANOVA.

| Age  Stages (years) | FTIR | | | | Raman | | | |
| --- | --- | --- | --- | --- | --- | --- | --- | --- |
|  | Mineral/matrix | Carbonate/phosphate | Crystallinity | Collagen maturity | Mineral/matrix | Carbonate/phosphate | Crystallinity | Collagen maturity |
| 0~3 | 0.572 | 0.791 | 0.739 | 0.172* | 0.433 | 0.105 | 0.600* | 0.248* |
| 3~12 | 0.210 | 0.698 | 0.586 | 0.767 | 0.883 | 0.144 | 0.674 | 0.739 |
| 12~19 | 0.674 | 0.933 | 0.861 | 0.092 | 0.249 | 0.970 | 0.591 | 0.932 |
| 19~35 | 0.164 | 0.501 | 0.602* | 0.566 | 0.740 | 0.002 | 0.841 | 0.263 |
| 35~60 | 0.986* | 0.766 | 0.651 | 0.418* | 0.638 | 0.072 | 0.636 | 0.545 |
| >60 | 0.393 | 0.802 | 0.430 | 0.917* | 0.617 | 0.251 | 0.838 | 0.906 |


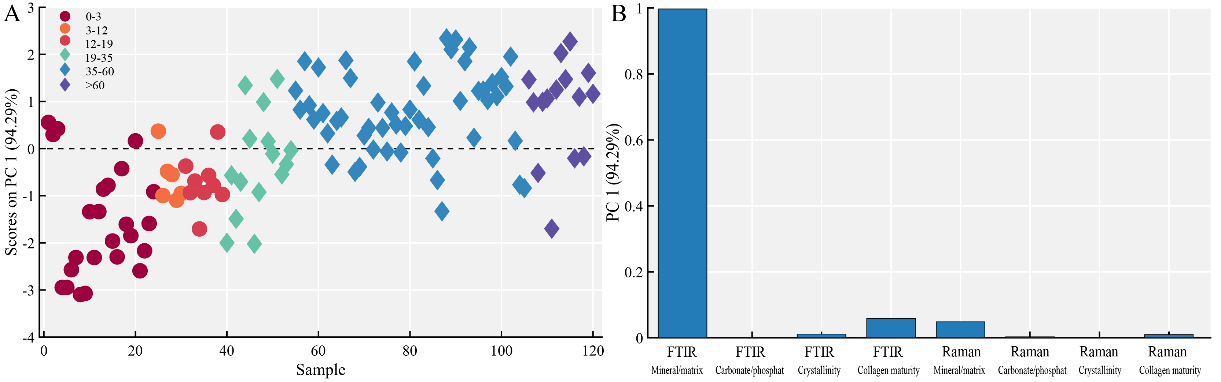


Fig. S1 The PCA results of eight widely used FTIR and Raman outcomes. **A** PCA score plot for 6 age stages. **B** PC1 loading plot.


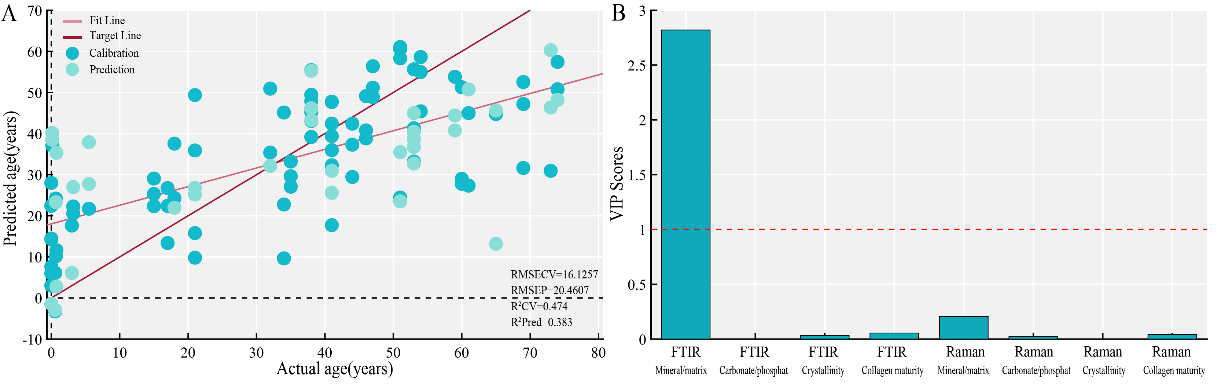


Fig. S2 **A** PLS regression model with the eight outcomes of FTIR and Raman. **B** PLS regression model VIP scores plot.
