# Supplementary material for: Chronological Age Estimation of Male Occipital Bone Based on FTIR and Raman Microspectroscopy
Source: Bioinorg Chem Appl. 2022 Aug 26;2022:1729131. doi: 10.1155/2022/1729131 (PMC9440630; doi:10.1155/2022/1729131)
Supplement: Supplementary Materials — Table S1: FTIR and Raman statistical results of different structures (lamina externa, diploe, and lamina interna) at each age stages. ∗The results of Kruskal–Wallis test that failed to exhibit normal distributions or equal variance. And the rest are the results of Single-factor ANOVA. Figure S1: The PCA results of eight widely used FTIR and Raman outcomes. A PCA score plot for 6 age stages. B PC1 loading plot. Figure S2: A PLS regression model with the eight outcomes of FTIR and Raman. B PLS regression model VIP scores plot. [file 1729131.f1.zip › 1729131.f1/Supplementary Material .pdf]

Chronological age estimation of male occipital bone based on FTIR and Raman microspectroscopy

Kai Yu[1], Hongli Xiong[3], Xin Wei[1], Hao Wu[1], Bo Zhang[4], Gongji Wang[1], Xiaorong Yang[2]*, Zhenyuan Wang[1]*

[1]Department of Forensic Pathology, College of Forensic Medicine, Xi'an Jiaotong University, Xi'an 710061, PR China

[2]Department of Forensic Medicine, Guiyang Medical University, Guiyang, Guizhou, 550025, PR China

[3] Department of Forensic Medicine, Faculty of Basic Medical Sciences, Chongqing Medical University, Chongqing 400016, PR China

[4] Xi'an Jiaotong University, Xi'an 710061, PR China

*Corresponding author: Zhenyuan Wang, PhD, Department of Forensic Pathology, College of Forensic Medicine, Xi'an Jiaotong University, Xi'an 710061, PR China. Phone: +8629 13709110298. E-mail: wzy218@xjtu.edu.cn.

Xiaorong Yang, MD, Department of Forensic Medicine, Guiyang Medical University, Guiyang, Guizhou, 550025, PR China. Phone: +86851 18085148797. E-mail: fyyxr_820613@163.com.

Table S1 FTIR and Raman statistical results of different structures (lamina externa, diploe and lamina interna) at each age stages. *The results of Kruskall-Wallis test that failed to exhibit normal distributions or equal variance. And the rest are the results of Single-factor ANOVA.

Age Stages (years)	FTIR Mineral/matrix	FTIR Carbonate/phosphate	FTIR Crystallinity	FTIR Collagen maturity	Raman Mineral/matrix	Raman Carbonate/phosphate	Raman Crystallinity	Raman Collagen maturity
0~3	0.572	0.791	0.739	0.172*	0.433	0.105	0.600*	0.248*
3~12	0.210	0.698	0.586	0.767	0.883	0.144	0.674	0.739
12~19	0.674	0.933	0.861	0.092	0.249	0.970	0.591	0.932
19~35	0.164	0.501	0.602*	0.566	0.740	0.002	0.841	0.263
35~60	0.986*	0.766	0.651	0.418*	0.638	0.072	0.636	0.545
>60	0.393	0.802	0.430	0.917*	0.617	0.251	0.838	0.906

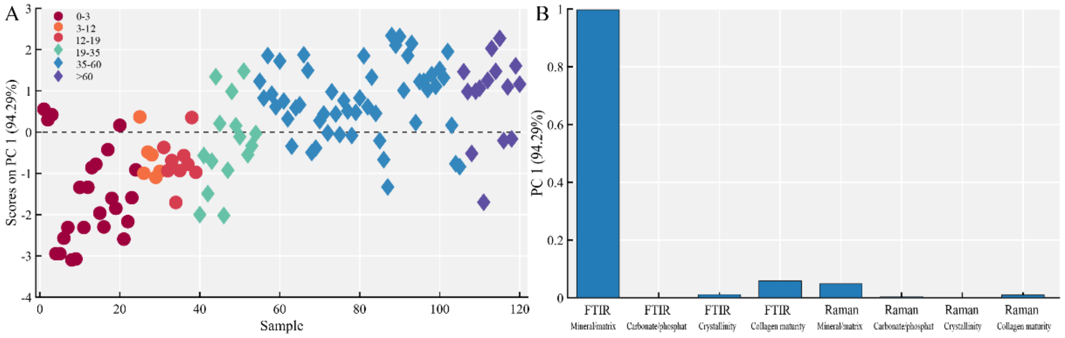

Fig. S1 The PCA results of eight widely used FTIR and Raman outcomes. **A** PCA score plot for 6 age stages. **B** PC1 loading plot.

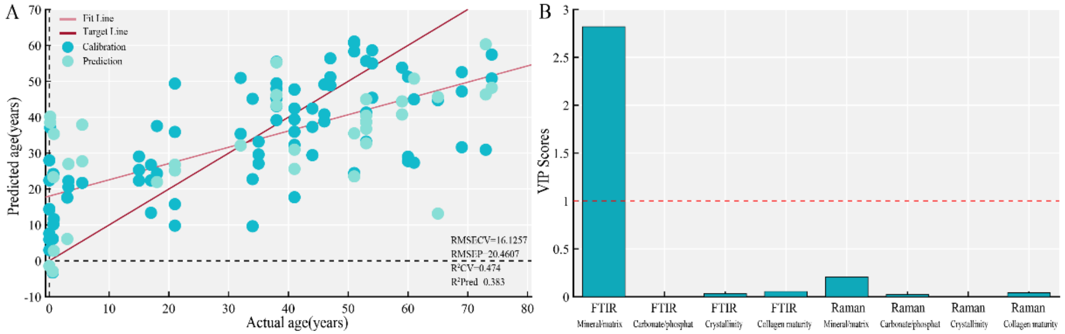

Fig. S2 **A** PLS regression model with the eight outcomes of FTIR and Raman. **B** PLS regression model VIP scores plot.
